# Differential effect of interferon-alpha treatment on AEA and 2-AG levels

**DOI:** 10.1016/j.bbi.2020.08.024

**Published:** 2020-11

**Authors:** Zuzanna Zajkowska, Alessandra Borsini, Naghmeh Nikkheslat, Alice Russell, Graziella F. Romano, Simona Tomassi, Nilay Hepgul, Daniel Forton, Kosh Agarwal, Matthew Hotopf, Valeria Mondelli, Patricia Zunszain, Carmine M. Pariante

**Affiliations:** a Department of Psychological Medicine, Institute of Psychiatry, Psychology and Neuroscience, Kings College London, UK; b Department of Gastroenterology & Hepatology, St George’s University of London, UK; c Institute of Liver Studies, King’s College Hospital, UK; d National Institute for Health Research (NIHR), Mental Health Biomedical Research Centre at South London and Maudsley NHS Foundation Trust and King’s College London, UK

**Keywords:** Endocannabinoid system, Anandamide (AEA), 2-Aracgidonoylglycerol (2-AG), Inflammation, Cytokines, Immune system, Hepatitis C, Interferon-α (IFN-α), IFN-α-induced depression, Depression

## Abstract

The endocannabinoid (eCB) system is one of the key players in immunoregulation, and reduced activity of the eCB system has been linked with depressive-like behaviours in animal studies and depression in clinical samples. There is a well-established link between immune activation and depression, such as following the administration of the pro-inflammatory cytokine, interferon-α (IFN-α), used to treat hepatitis C viral (HCV) infection. However, the role of peripheral endocannabinoids (eCBs), anandamide (AEA) and 2-arachidonoylglycerol (2-AG), following immunotherapy with IFN-α and in IFN-α -induced depression, have not been examined yet.

In this study, we investigated whether circulating AEA and 2-AG were modified by treatment with IFN-α and whether they were involved in the development of IFN-α-induced depression. We also explored whether circulating eCBs were associated with peripheral cytokines during and after IFN-α treatment. We measured serum concentrations of AEA and 2-AG using High Performance Liquid Chromatography with Tandem Mass Spectrometry, and serum concentrations of cytokines using Meso Scale Discovery electrochemiluminescence V-PLEX assay, in 70 patients with HCV infection and 41 healthy subjects. We assessed HCV patients at baseline, IFN-α-treatment weeks (TW) 4 and 24, end of treatment (END) and at six months follow-up (FU). We assessed depression using M.I.N.I. International Neuropsychiatric Interview.

We found a different pattern of change in peripheral AEA and 2-AG during and after IFN-α treatment. Whilst 2-AG increased earlier in immunotherapy (TW4), remained elevated throughout treatment, and reduced at six months follow-up (FU), AEA increased later in treatment (TW24) and remained elevated six months post-treatment. We also found that baseline levels of AEA were lower in HCV patients compared with healthy controls, whereas there were no differences in 2-AG levels. Interestingly, AEA, but not 2-AG, was significantly, negatively correlated with interleukin (IL)-2 and IL-17a at six months follow-up. We did not find any difference in both eCBs between patients with and without IFN-α-induced depression, at any time point.

Our findings suggest that AEA and 2-AG are involved in different stages of immunoregulation following IFN-α treatment, where AEA might be involved in chronic inflammation. Lack of association between peripheral eCBs and IFN-α-induced depression suggests that different biological mechanisms may underpin inflammation-induced depression compared with classic “psychiatric” depression, or that any changes in the eCB system in depression may not be captured by peripheral AEA and 2-AG.

## Highlights

• AEA and 2-AG play part in different stages of IFN-α induced immune response.
• AEA might be acting in the immunoregulation of chronic inflammation.
• 2-AG may be involved in the initial stages of inflammatory response.
• Targeting eCB signalling may be of interest as an anti-inflammatory treatment.

## 1 Introduction

Considerable research suggests a bi-directional interaction between the endocannabinoid (eCB) and immune systems, with the eCB system being involved mainly in attenuation of inflammation through the regulation of both, the innate and adaptive immune systems (Boorman et al., 2016). However, most of the studies derive from preclinical models and data in humans are lacking (Abidi et al., 2018, Fechtner et al., 2019, Fitzgibbon et al., 2019). The eCB system is a homeostatic signalling system with inhibitory properties identified following the discovery of the main psychoactive constituent of Cannabis Sativa *L*., Δ^9^-tetrahydrocannabinol (THC) (Gaoni & Mechoulam, 1964). This system consists of three main components: 1) the endogenous lipid transmitters called endocannabinoids (eCBs), the main ones being anandamide (AEA) and 2-arachidonoylglycerol (2-AG) (Devane et al., 1992, Mechoulam et al., 1995); 2) the target of these eCBs, the G-protein-coupled receptors, cannabinoid receptor type 1 (CB1R) and type 2 (CB2R) (Matsuda et al., 1990, Munro et al., 1993); and 3) enzymes involved in the synthesis of AEA and 2-AG, namely N-acyl phosphatidylethanolamine phospholipase D (NAPE-PLD) and diacylglycerol lipase (DAGL) (Bisogno et al., 2003, Okamoto et al., 2004) respectively, and in the degradation of AEA and 2-AG, fatty acid amide hydrolase (FAAH) and monoacylglycerol lipase (MAGL), respectively.

Evidence suggests an involvement of eCB signalling primarily in decreasing inflammation (Crowe et al., 2014). For instance, CB2R activation has been shown to attenuate inflammation in a range of inflammatory conditions including injury, inflammatory pain, hepatic injury, periodontitis, multiple sclerosis, rheumatoid arthritis and intestinal inflammatory disorders (Abidi et al., 2018, Correa et al., 2011, Fechtner et al., 2019, Pandey et al., 2009). Furthermore, levels of circulating protein and mRNA encoding for interleukin- (IL)-1, IL-6 and tumor necrosis factor (TNF)-α are reduced by the administration of synthetic cannabinoids in preclinical models of multiple sclerosis and periodontitis (Abidi et al., 2018, Croxford and Miller, 2003). In addition, in an animal model of autoimmune hepatitis the administration of AEA has been shown to diminish hepatic injury, and this is correlated with a significant reduction in inflammatory cytokines, such as TNF-α, IL-1β, IL-6, IL-9 and IL-17. The therapeutic effects of AEA are CB1R and CB2R dependent, as blockade of the receptors independently inhibits the anti-inflammatory effects (Hegde et al., 2008). Several types of immune cells produce and regulate eCB activity, including macrophages, basophils, lymphocytes and dendritic cells (Bisogno et al., 1997, Liu et al., 2003, Maccarrone et al., 2001). For instance, rat macrophage and basophil cells produce and degrade AEA (T Bisogno et al., 1997). In humans, after one year of treatment with interferon- (IFN)-β in multiple sclerosis, levels of AEA in natural killer (NK), T and B cells and expression of CB1R and CB2R in T and B cells, progressively decreased (López et al., 2014).

The eCB system is also implicated in depression (Crowe et al., 2014, Poleszak et al., 2018). Animal models suggest reduced functioning of the system across receptor, enzyme and circulating eCBs activity (Fang and Wang, 2018, Poleszak et al., 2018). Clinical investigations of eCB-associated effects in depression are relatively sparse. Nonetheless findings have, for the most part, verified the association between reduced eCB activity and depression. For example, Rimonabant, a CB1R antagonist developed for use as an anti-obesity drug, was withdrawn from the market due to serious adverse effects on mood that resulted in onset of depression and suicidal ideation (Topol et al., 2010). Another study reported that patients undergoing cardiac surgery who developed depression six-months post operation, had significantly reduced plasma levels of AEA and 2-AG during perioperative period (Hauer et al., 2012). Furthermore, observational studies have shown reduced serum levels of AEA and 2-AG in depressed women compared with healthy controls, in one of which a reduction in serum 2-AG progressively decreased over the duration of the disorder (Hill et al., 2009, Hill et al., 2008). Interestingly, another study found that patients with depression who received selective serotonin reuptake inhibitor (SSRI) treatment had elevated plasma levels of 2-AG compared with those who were not on antidepressant treatment, although this effect was not present for AEA levels (Romero-Sanchiz et al., 2019). The notion of reduced eCB function in depression is particularly interesting in view of the putative anti-inflammatory action of this system (discussed above), as there is a well-established link between increased inflammatory state and depression. In fact, several *meta*-analyses confirm the association between depression and increased inflammation, with depressed patients showing elevated levels of IL-6, IL-1, TNF-α and C-reactive protein (CRP) compared with healthy controls (Dowlati et al., 2010, Horn et al., 2018, Howren et al., 2009, Perrin et al., 2019). Some studies have further shown that increased peripheral inflammation might be associated with different subtypes of depression, including treatment resistant depression and specific traits such as anhedonia or motor and cognitive slowing (Haroon et al., 2018a, Haroon et al., 2018b, Raison et al., 2013).

Prospective cohort studies have also reported that increased inflammatory state frequently leads to development of depression. For example, in the most widely used model of inflammation-induced depression, up to 45% of patients receiving pegylated- (peg)-IFN-α and Ribavirin for hepatitis C virus (HCV) develop depression during the course of treatment, alongside with an increase in levels of cytokines and immune genes expression (Hepgul et al., 2016, Machado et al., 2017, Perrin et al., 2019). However, no studies so far, explored the role of eCBs in inflammation-induced depression.

In this study, we focused on the role of peripheral eCBs, AEA and 2-AG, in patients with chronic HCV infection receiving peg-IFN-α treatment, the aforementioned immune challenge and established model to investigate inflammation-induced depression using a longitudinal design. We specifically measured changes in serum AEA and 2-AG before and during IFN-α treatment (treatment weeks 4 and 24), at the end of treatment (END), and six-months post-treatment, and investigated whether these changes were associated with IFN-α induced depression. We also measured peripheral cytokines to monitor the inflammatory changes during and after IFN-α treatment, and their co-variance with eCB levels. Finally, we looked at the relationship between the eCB and immune systems six-months post-treatment with IFN-α, to measure any potential recovery of eCB levels after the immune challenge was no longer present. The comparison in eCB levels between patients with HCV and healthy controls was also examined, since one previous study found that plasma AEA and 2-AG were elevated in HCV patients compared with the control group (Patsenker et al., 2015). To our knowledge, this is the first study to use a prospective cohort design in order to explore the role of peripheral eCBs, in both, patients with HCV receiving IFN-α treatment, and IFN-α induced depression.

## 2 Materials and methods

The study was approved by the South East London Research Ethics Committee 3 (REC ref:10/H0808/30) and London Dulwich Research Ethics Committee (REC ref: 12/LO/1368).

### 2.1 Participants

We recruited 70 patients with chronic HCV infection and compensated liver disease, due to receive their treatment, from the Institute of Liver Studies at King’s College Hospital in London, the Gastroenterology Departments at St George’s, Queen Mary’s (Roehampton), St Thomas’ and Royal Free Hospitals in London. The age range of the HCV sample was 18–68 years old. The treatment consisted of weekly, subcutaneous injection of peg-IFN-α 2-b (1.5 mg/Kg) and daily Ribavirin (rib), administered orally in doses ranging from 800 to 1400 mg. In addition to IFN-α/rib, nine subjects (13%) were also prescribed a direct-acting antiviral – ‘triple therapy’, adding boceprevir n = 1, telaprevir n = 4 or simeprevir n = 2 (missing values n = 2), after treatment protocols changed across the UK.

Exclusion criteria included presence of depression at baseline, use of antidepressants at baseline, active substance abuse, pregnancy, any autoimmune conditions, acute infections, or other than HCV causes for liver disease, and language barrier (requiring interpreter). Clinical and biological assessments were performed at treatment week (TW) 0 (baseline), TW4, TW24, and at the end of treatment (END) which combined TW24, 36 or 48 depending on the duration of treatment (TW24-81.43%, TW36-7.14% and TW48-11.43%). Then, patients were followed up six-months after the end of treatment. A total of 28 patients (40%) developed depression at some point during IFN-α treatment, whereas 42 patients (60%) did not develop depression. The attrition rate at six months follow-up (FU) was 40%. There were no significant differences between patients who dropped out compared with those who did not, in age, gender, current tobacco and cannabis use, viral load, fibroscan score or viral genotype (data not shown).

Healthy controls (N = 41), matched for age and gender, were recruited from the community and from within King’s College London – staff and students. The age range of healthy controls was 21–67 years old. The inclusion criteria were as follows: no significant health conditions, not taking any regular medication in the last 3 months prior to the assessment (excluding contraception), not experienced a recent or recurrent episodes of mental illness, no past or present substance abuse or dependency. Healthy volunteers attended one visit at the Clinical Research Facility at King’s College Hospital where all the clinical and biological assessments were conducted. The sample was partially overlapping with that in the publication by our colleagues (Hepgul et al., 2016, Russell et al., 2019).

### 2.2 Clinical measures

We used the Mini International Neuropsychiatric Interview (MINI) Major Depression section to assess the depression at baseline and the development of IFN-α-induced depression in HCV patients during treatment (TW4, 24 and END), and at six months follow-up. We used the same measure to assess past and current depression in healthy volunteers (Sheehan et al., 1998). Other measures administered at baseline included modified version of the Cannabis Experience Questionnaire to assess cannabis, tobacco and alcohol use (Barkus et al., 2006), and Socio-Demographic Schedule (SDS) to record age, gender, ethnicity, educational level, employment and relationship status (Mallett et al., 2002). Demographic data are shown in Table 1.

**Table 1:** Sample characteristics.

*Treatment week (TW).

### 2.3 HCV measures

We obtained the HCV RNA viral load from the patient medical records to assess the severity of the illness at baseline as well as treatment response. The viral load was measured by the number of viral particles per ml of blood, presented in millions (AmpiliPrep, Roche), where a result of <15 IU/ml equated to undetected. The viral load was measured at treatment week 4 to assess Rapid Virological Response (undetected virus at week 4 indicates likely treatment success), and six months post-treatment to assess Sustained Virological Response which determined treatment outcome. We also examined the degree of liver damage by assessing liver stiffness reported in the Fibroscan result at baseline. See Table 1 for full results on HCV measures.

### 2.4 Biological markers

In the HCV sample biological assessments were also conducted at treatment weeks 0, 4, and 24, at the end of treatment, and at six months follow-up. Due to the sample attrition, we were only able to collect data from 42 patients at the six months follow-up. In the control group, we performed all the biological assessments at the one-off visit. Blood samples were collected using 6 mL BD vacutainer plastic tubes (silica clot activator). Samples were left to clot for minimum 30 min at room temperature and then centrifuged at 1850g for 10 min at room temperature, following which, serum was removed and frozen at −80 °C. We adjusted for the missing data by selecting only those samples which had all the time points completed.

### 2.5 Endocannabinoids

AEA and 2-AG concentrations were analysed using High Performance Liquid Chromatography (HPLC) with tandem mass spectrometry (MS/MS) detection. Samples were precipitated 1:4 with 80% methanol, then vortexed and centrifuged at 16000×*g* for 5 min. The internal standards D_4_-AEA and D_5_-2-AG (Cayman Chemicals, Ann Arbor, MI) were added to the supernatant. Concentrations of AEA and 2-AG were determined by HPLC with tandem mass spectrometry (MS/MS) detection, using D_4_-AEA and D_5_-2-AG as internal standards for the analytes. 27 μL of each sample was injected onto the HPLC column by an automated sample injector (SIL-10-20AC-HT, Shimadzu, Japan). Chromatographic separation was performed on a reversed phase column (100 × 2.1 mm, particle size 2.6 µm; Thermo), held at a temperature of 45 °C. The mobile phases consisted of A: methanol:ultrapurified H_2_O (50:50) + 0.1% formic and B: methanol:ultrapurified H_2_O (90:10) + 0.1% formic acid. Elution of the compounds proceeded using a suitable linear gradient at a flow rate of 0.25 mL/min over 9 min. The MS analyses were performed using an API 5000 MS/MS system consisting of an API 5000 MS/MS detector and a Turbo Ion Spray interface (Applied Biosystems, the Netherlands). The acquisitions on API 5000 were performed in positive ionization mode, with optimized settings for the analytes (AEA: *m*/*z* 379 ->269; 2-AG: *m*/*z* 348 ->287). The instrument was operated in multiple-reaction-monitoring (MRM) mode. Data were calibrated and quantified using the Analyst data system (Applied Biosystems, version 1.4.2, the Netherlands). Concentrations in experimental samples were calculated based on the calibration curve in the corresponding matrix. The calibration curves ranged from 0.025 nM to 20 nM for AEA and from 0.25 to 200 nM for 2-AG (both from Cayman Chemicals, Ann Arbor, MI).

### 2.6 Cytokines

Meso Scale Discovery (MSD) electrochemiluminescence V-PLEX assay was used to measure cytokines including IL-2, IL-6, IL-4, IL-8, IL-10, IL-13, TNF-α, IL-7 and IL-17a (pg/mL). Serum samples were diluted 2-fold and measured in duplicate. The cytokines concentration was determined with the electrochemiluminescent labels whilst the plate is inserted into the MSD instrument (MESO QUICKPLEX SQ 120). All samples were assayed in duplicate. High and low controls were used to assess variance between plates. The inter-assay coefficient of variations was <10%. The results were analysed using MSD DISCOVERY WORKBENCH analysis software.

### 2.7 Data analysis

All data were analysed with IBM SPSS statistical software version 22. For the graphic representation of the data, unless otherwise stated, the mean score is presented with error bars representing the standard error of the mean (SEM). The significance value for all tests was set at α = 0.05. To examine differences between two groups in continuous variables, independent samples t-tests were used, where data was normally distributed. Where equal variance could not be assumed, as assessed by the Levene’s test (*p* < 0.05), the results reported are those where the degrees of freedom have been adjusted using the Welch-Satterthwaite method. Where data was not normally distributed, a post-hoc Mann-Whitney *U* test of difference was used. To measure changes in eCB levels during and after treatment, and differences in eCB levels between patients with and without IFN-α induced depression, we used two-way mixed ANOVA model followed by the Bonferroni correction for multiple comparisons. To measure changes in cytokine levels during and after treatment in the HCV sample, we used one-way repeated measures ANOVA model followed by the Bonferroni correction for multiple comparisons. Where data were not normally distributed, logarithmic transformations of the data were used. Where skewness was addressed successfully, log transformed analysis was reported, and post-hoc analyses were performed using parametric tests. Where the attempts to transform the data proved unsuccessful, analysis was performed on the raw data, and post-hoc analyses were performed using appropriate non-parametric test. Where the assumption of sphericity was violated (*p* < 0.05) as indicated by Mauchly’s test, results reported are those with the Greenhouse-Geisser [G-G] correction to the degrees of freedom applied. Associations between two continuous variables were explored using Pearson’s product moment correlations (Pearson’s r; *r*), where data for both variables was linear and normally distributed. Where data was not linear, log transformations were performed and analyses run on transformed data. Where data was not normally distributed Spearman’s rank correlation co-efficient (Spearman’s Rho; *r_s_*) was used.

## 3 Results

### 3.1 IFN-α treatment increases serum AEA and 2-AG in patients with HCV

**Fig. 1:**
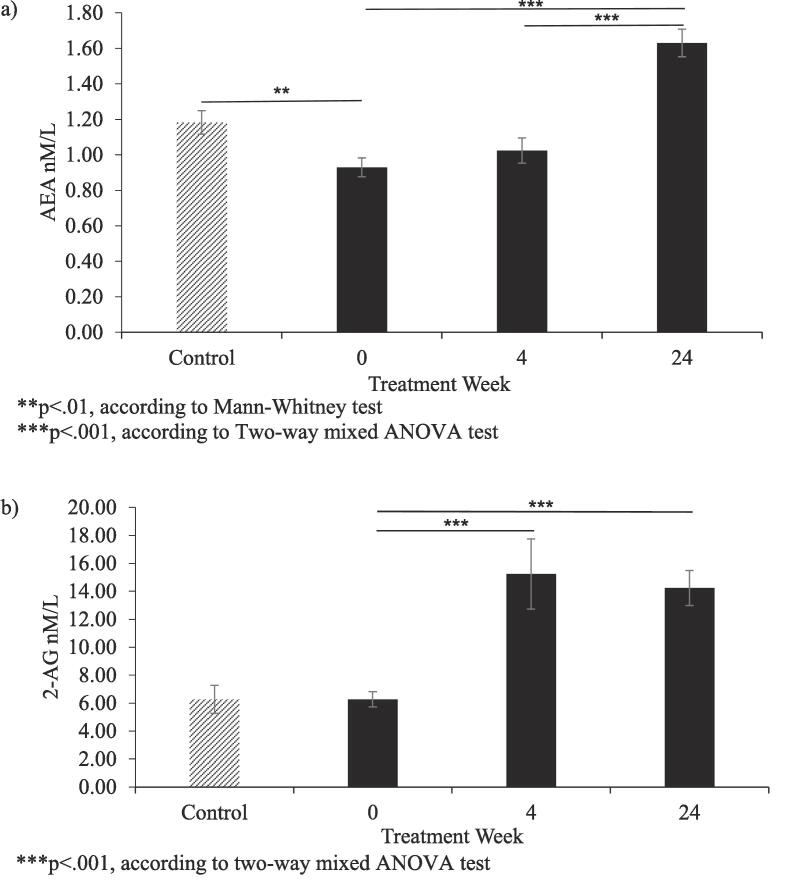
Panel a) AEA levels in HCV patients during IFN-α treatment presented at baseline and at treatment weeks 4 and 24, and in healthy controls at baseline; panel b) 2-AG levels in HCV patients during IFN-α treatment presented at baseline and at treatment weeks 4 and 24, and in healthy controls at baseline.

We first analysed the peripheral AEA and 2-AG levels before IFN-α treatment, in patients with HCV and healthy controls. We found that HCV patients showed significantly lower baseline serum AEA levels compared with healthy controls (U = 1989.5, z = 3.39, p = .001). No significant difference was found for baseline serum 2-AG between patients and controls (t (1 0 9) = 0.001, p = .99) (see Fig. 1).

Then, we looked at changes in AEA and 2-AG levels in patients with HCV during IFN-α treatment. Although both AEA and 2-AG increased significantly during treatment, the pattern of change was different for each eCB (AEA: F (2, 136) = 73.66, p < .001, partial n^2^ = 0.52; 2-AG: F (1.27, 87.35) = 10.32, p = .001, partial n^2^ = 0.13). Whilst 2-AG increased significantly at TW4 (p = .001) and remained elevated until TW24, AEA increased later during treatment, at TW24 (p < .001) (see Fig. 1).

**Fig. 2:**
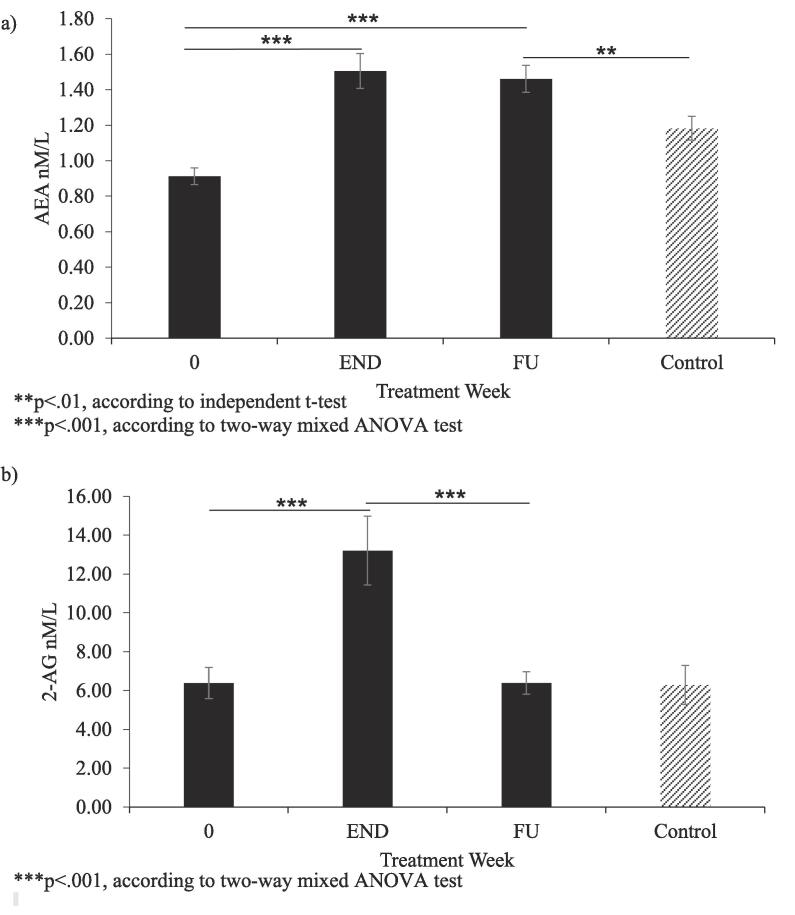
Panel a) AEA levels after IFN-α treatment in HCV patients presented at baseline, end of treatment (END) and at six months follow-up (FU), and in healthy controls at baseline; panel b) 2-AG levels after IFN-α treatment in HCV patients presented at baseline, end of treatment (END) and at six months follow-up (FU), and in healthy controls at baseline.

Finally, we looked at changes in AEA and 2-AG levels between end of treatment and six-months post-treatment. These analyses were conducted separately due to the sample being smaller (n = 42 vs. n = 70) as follow-up serum was not available for everybody. In addition, we also compared follow-up values with those of healthy controls, as by then most (n = 33 out of n = 42) HCV patients had responded to the antiviral therapy. In these analyses, AEA remained elevated at follow up, even in the absence of IFN-α (F (2, 78) = 34.19, partial n^2^ = 0.47, p < .001) and remained elevated even when compared with healthy controls (t (81) = 2.740, p = .008). In contrast, 2-AG levels reduced at follow-up compared with end of treatment (F (1.34, 52.4) = 15.61, partial n^2^ = 0.29, p < .001) and were not significantly different from controls (U = 710.500, z = -1.379, p = .17) (see Fig. 2).

### 3.2 Serum AEA and 2-AG in IFN-α induced depression

Firstly, we explored the difference in peripheral AEA and 2-AG levels at baseline, between patients who went on to develop depression and patients who did not develop depression during IFN-α treatment. We performed this analysis to understand whether eCBs can be used as potential predictors of IFN-α induced depression. We did not find any significant difference in AEA and 2-AG levels at baseline between patients with and without IFN-α induced depression (AEA: t (68) = −0.035, p = .97; 2-AG: t (68) = 1.605, p = .11).

**Fig. 3:**
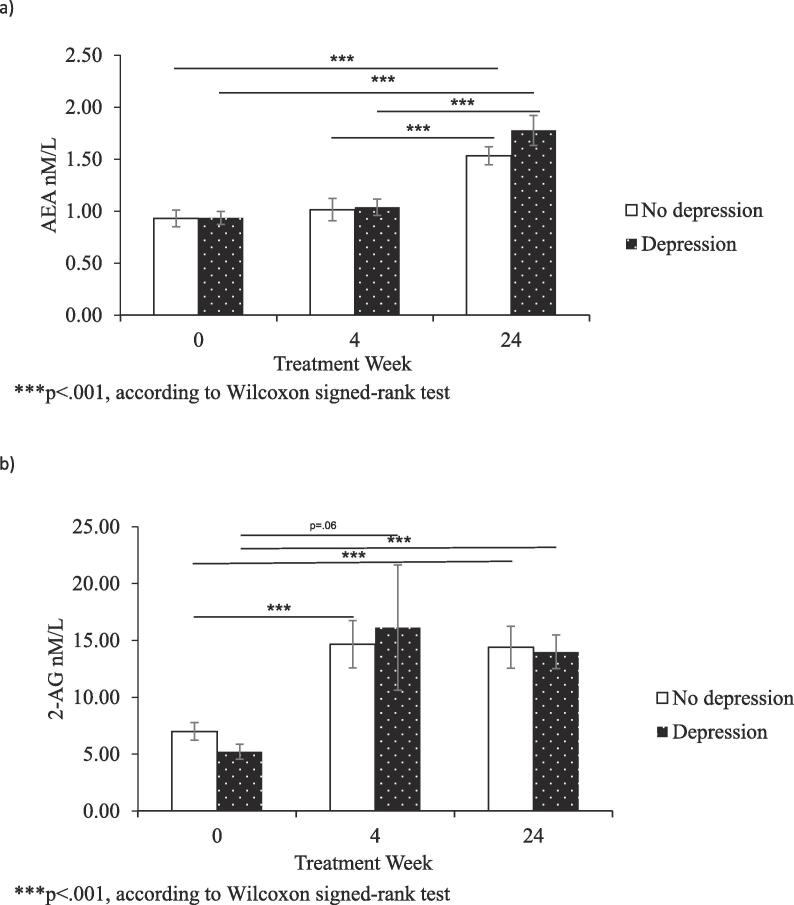
Panel a) AEA levels in HCV patients with and without onset of depression during IFN-α treatment presented at baseline and at treatment weeks 4 and 24; panel b) 2-AG levels in HCV patients with and without onset of depression during IFN-α treatment presented at baseline and at treatment weeks 4 and 24.

**Fig. 4:**
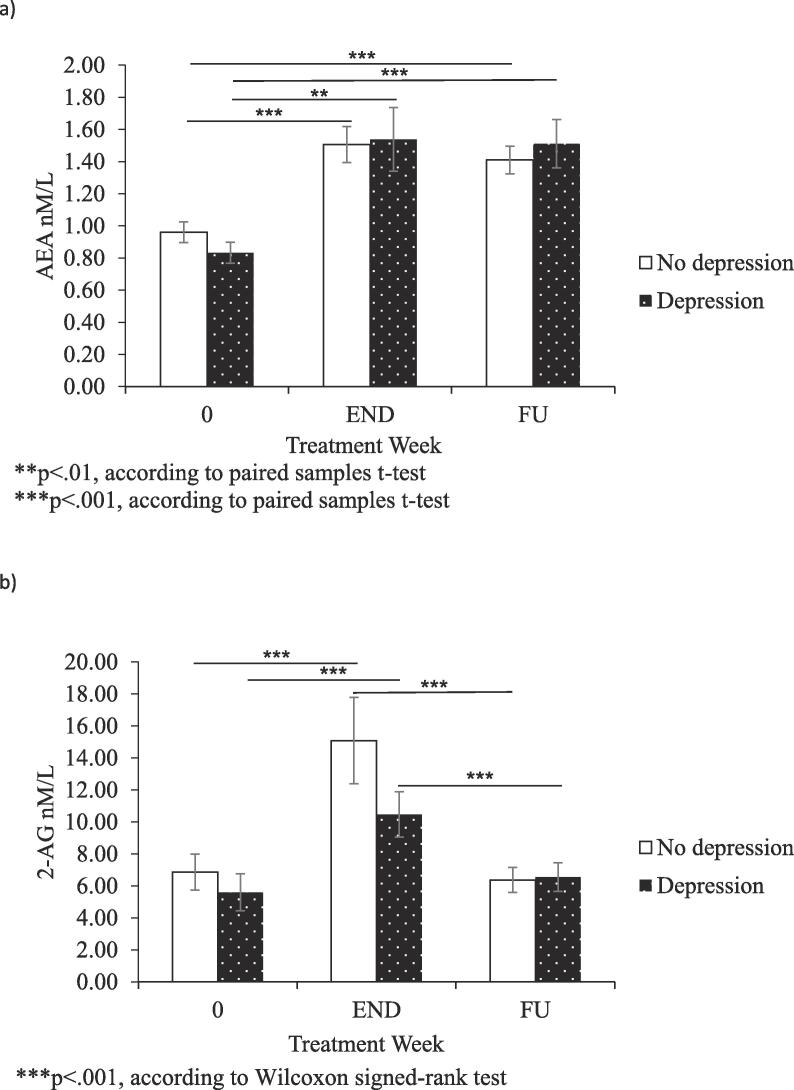
Panel a) AEA levels in HCV patients with and without onset of depression after IFN-α treatment presented at baseline, end of treatment (END) and at six months follow-up (FU); panel b) 2-AG levels in HCV patients with and without onset of depression after IFN-α treatment presented at baseline, end of treatment (END) and at six months follow-up (FU).

Subsequently, we analysed the changes in AEA and 2-AG levels during IFN-α treatment and six-months post-treatment in patients with and without IFN-α induced depression. We found no significant differences nor group × time interactions in AEA and 2-AG levels between depressed and non-depressed patients during (AEA main group effect: F(1, 68) = 1.169, p = 0.28, partial n^2^ = 0.17; AEA group × time: F (2, 136) = 0.211, p = 0.81, partial n^2^ = 0.003; 2-AG main group effect: F(1, 69) = 0.017, p = .89, partial n^2^ = 0.006; 2-AG group × time: F (1.266, 87.350) = 0.347, p = .61, partial n^2^ = 0.005) and after IFN-a treatment (AEA main group effect: F(1, 39) = 0.000, p = 0.99, partial n^2^ = 0.91; AEA group × time: F (2, 78) = 0.993, p = 0.38, partial n^2^ = 0.25; 2-AG main group effect: F(1, 39) = 0.955, p = .33, partial n^2^ = 0.24; 2-AG group × time: F (1.344, 52.403) = 1.718, p = 0.19, partial n^2^ = 0.042). These results are presented in Fig. 3, Fig. 4. The data indicate that the increase in AEA and 2-AG we observed is independent of depression development.

### 3.3 Association between peripheral inflammation and endocannabinoids during and after IFN-a treatment in HCV patients

We wanted to understand the relationship between eCBs and cytokines during the immune activation following IFN-α treatment. To do that, we measured cytokines in the HCV patients during treatment and six months post-treatment (these cytokines data on a larger, overlapping sample, have already been presented by our group in Russell et al., 2019). To limit the number of multiple comparisons, we selected the cytokines that significantly changed during this period and correlated them with eCB levels measured at the same time point. During IFN-a treatment, HCV patients showed significant increase in serum IL-2, IL-6, IL-17a and TNF-a (all at weeks 4 and 24), and decrease in serum IL-4 (at week 4). At six months post-treatment, HCV patients showed a significant decrease in serum IL-2, IL-6, IL-10, IL-17a and TNF-α compared with end of treatment, and all cytokines at this time point were no longer different from their baseline levels, except for IL-10, which was lower than its baseline levels (see Table 2). Given that all cytokines affected by IFN-a treatment decreased at six months follow-up, we also explored if they were associated with the significantly elevated levels of AEA at six months follow-up.

**Table 2:** Cytokine changes during and after IFN-α treatment.

Measure	HCV TW0	HCV TW4	HCV TW24	HCV FU	Cytokine changes during and after IFN-α treatment (TW0, 4, 24 and FU)*
IL-2	0.4 ± 0.12	0.46 ± 0.12	0.54 ± 0.13	0.4 ± 0.13	F(3, 108) = 13.329, p < .001, partial n^2^ = .27 (**TW0 > TW4, p = .04; TW0 > TW24**,**p < .001**; TW0 > FU, p = 1)
IL-4	0.04 ± 0.01	0.021 ± 0.007	0.02 ± 0.005	0.04 ± 0.01	F(2.22, 79.91) = 4.054, p = .02, partial n^2^ = .1 (**TW0 > TW4, p = .04**; TW0 > TW24, p = .19; TW0 > FU, p = 1)
IL-6	0.95 ± 0.16	1.67 ± 0.25	1.87 ± 0.28	1.09 ± 0.16	F(3, 108) = 16.5, p < .001, partial n^2^ = .31 (**TW0 > TW4, p < .001; TW0 > TW24, p < .001**; TW0 > FU, p = .93)
IL-7	15.95 ± 1.44	16.83 ± 1.76	17.72 ± 1.6	17.49 ± 2.17	F(3, 108) = 1.408, p = .25, partial n^2^ = .04
IL-8	26.61 ± 7.78	28.29 ± 6.52	29.69 ± 6.93	16.43 ± 3.42	F(2.522, 90.786) = 3.455, p = .03, partial n^2^ = .09 (TW0 > TW4, p = 1; TW0 > TW24, p = 1; TW0 > FU, p = .48)
IL-10	1.1 ± 0.24	1.02 ± 0.14	1.09 ± 0.17	0.54 ± 0.09	F(2.531, 91.132) = 18.894, p < .001, partial n^2^ = .34 (TW0 > TW4, p = 1; TW0 > TW24, p = 1; **TW0 > FU, p < .001)**
IL-13	0.48 ± 0.12	0.49 ± 0.12	0.51 ± 0.12	0.49 ± 0.12	F(3, 108) = .736, p = .53, partial n^2^ = .02
IL-17a	2.01 ± 0.39	3.96 ± 0.97	3.44 ± 0.62	2.39 ± 0.55	F(3, 108) = 17.321, p < .001, partial n^2^ = .33 (**TW0 > TW4, p < .001; TW0 > TW24, p < .001**; TW0 > FU, p = 1)
TNF-α	4.53 ± 0.41	6.09 ± 0.45	7.04 ± 0.61	4.54 ± 0.42	F(2.159, 77.713) = 23.498, p < .001, partial n^2^ = .4 **(TW0 > TW4, p < .001; TW0 > TW24,**p < .001; TW0 > FU, p = 1)

*Treatment week (TW); six months follow-up (FU).

We found no significant correlations between all selected cytokines and both eCBs (AEA and 2-AG) during IFN-a treatment. However, we found that six months post-treatment, AEA was moderately and negatively correlated with IL-2 (r = -0.44, p = .01) and IL-17a (r_s_ = -0.39, p = .02) levels, and there were trends for negative, weak correlations with IL-6 (r_s_ = -0.33, p = .06) and IL-10 (r = -0.31, p = .08) (see Table 3).

**Table 3:** Correlations between endocannabinoids and cytokines.

*Treatment week (TW); six months follow-up (FU).

^in bold significant values; in italic – trend for significance.

Lastly, we wanted to see whether any associations found were also present before IFN-a treatment. We found negative, weak correlation between AEA and IL-2 at baseline (r_s_ = −0.339, p = .03) and no significant correlation between baseline AEA and IL-17a (r_s_ = −0.01, p = .96) (see Table 3).

## 4 Discussion

We found that peripheral AEA and 2-AG were affected by HCV infection and IFN-α treatment. However, they were not associated with IFN-α induced depression. To our knowledge, this is the first study to use a prospective cohort design in order to explore the role of peripheral eCBs, in both, patients with HCV receiving IFN-α treatment, and IFN-α induced depression.

We demonstrated an increase in AEA and 2-AG levels during and after IFN-α treatment, albeit with a different pattern of change. We found that AEA increased at a later stage during treatment (TW24) and remained elevated even after treatment ended (six months follow-up). 2-AG on the other hand, increased earlier (TW4) and remained elevated during treatment, however, it returned to baseline levels six months post-treatment. This suggests that AEA and 2-AG might be involved in different stages of the immune response following IFN-α injection, where AEA continues playing part in more chronic inflammation. Previous research has shown that AEA participates in reducing inflammation while 2-AG can both, reduce and facilitate the inflammatory response (Chiurchiù et al., 2016, Gasperi et al., 2014, Hegde et al., 2008). For example, AEA reduced hepatic injury and the production of pro-inflammatory cytokines in autoimmune hepatitis (Hegde et al., 2008), and it was shown to suppress T cell proliferation and reduce cytokine production in several other studies (Cencioni et al., 2010, Chiurchiù et al., 2016, Schwarz et al., 1994). In contrast, 2-AG, has been shown to promote leukocyte recruitment by increasing the expression of adhesion molecules and TNF-α levels, which help leukocyte migration to the injury site (Gasperi et al., 2014). Furthermore, 2-AG has been found to increase levels of nitric oxide, a component of the inflammatory response (Chang et al., 2001). In another study, the inflammatory stimulation of human platelets and mouse macrophages with platelet activating factor (PAF) resulted in the immediate release of 2-AG but not AEA (Berdyshev, 2000). Differential regulation of AEA and 2-AG was also reported in response to lipopolysaccharide (LPS) in mouse macrophages, where only AEA levels increased following LPS stimulation, contributing to LPS-induced hypotension (Liu et al., 2003). Similarly, in another study only AEA levels were altered following LPS stimulation in the mice amygdala (Doenni et al., 2016). Although we might hypothesize, that the difference in the timing of increase between the two eCBs which we observe in our study might be due to different functions of AEA and 2-AG in the immune regulation, we cannot make strong statements due to the observational nature of our study.

In our sample, we did not find any difference in peripheral AEA and 2-AG in relation to IFN-α induced depression, before, during and after IFN-α treatment. Based on the previous literature, showing lower levels of eCBs in depression, we hypothesized that patients who developed depression at some stage during IFN-α treatment would have had lower baseline eCBs, making them more susceptible to depression, and that lower eCB levels would continue throughout treatment and beyond, in patients who develop depression (M. N. Hill et al., 2009). However, previous studies reporting eCB deficiency in depression did not investigate inflammation-induced depression, and it is possible that this “subtype” of depression is not associated with reduced eCBs (Doenni et al., 2016, Liu et al., 2003, Patsenker et al., 2015). As previous studies have shown, increased inflammation can lead to elevated eCB levels and elevated inflammation has been reported in a subgroup of depressed patients who are not responsive to antidepressant treatment (Doenni et al., 2016, Haroon et al., 2018b, Raison et al., 2013). This suggests that eCB levels might vary depending on the “subtype” of depression, albeit presence or absence of increased inflammation. The lack of association between eCB signalling and depressive-like behaviours in the presence of increased inflammation has been also reported in a preclinical model, where inhibition of the FAAH enzyme showed attenuation of neuroinflammation but no changes in depressive-like behaviour (Flannery et al., 2018). It is impossible to establish whether these two eCBs truly do not play any role in IFN-α induced depression (differently from “psychiatric” major depression), or simply we are not using the right biomarkers. In this regard, for example, we have shown, in a sub-sample of patients from this study, that patients who developed IFN-α-induced depression showed increased whole blood mRNA expression of immune markers which was not mirrored by an increase in circulating cytokines (Hepgul et al., 2016). This could be true for eCB markers as well, given the complex nature of this system (Hillard, 2017, Patsenker et al., 2015). Lastly, we are yet to understand whether peripheral eCB signalling mirrors eCB activity in the brain. We know from the current research that patients with psychiatric conditions, e.g., depression tend to have lower levels of circulating eCBs compared with healthy individuals, which suggests that there is a link between central nervous system (CNS) and peripheral eCBs (N. Hill et al., 2008, Hillard, 2017). Evidence from the recent study looking at eCBs in CNS showed increase in AEA levels in the cerebrospinal fluid of depressed patients following electroconvulsive therapy, which suggests that such form of antidepressant treatment resulted in elevated eCB signalling in the CNS. However, the same study reported no correlation between depressive symptoms decrease and AEA increase, and AEA levels were positively associated with depressive symptoms pre-treatment (Kranaster et al., 2017). We also know that peripheral eCBs can be transported across the blood–brain barrier (BBB) through endothelial cell membranes of brain microvessels (Boorman et al., 2016). However, we do not have sufficient evidence to say that circulating eCBs might reflect endocannabinoid levels in the brain (Hillard, 2017). To understand such correlation, more studies are needed looking at both, CNS and peripheral levels of eCBs.

Finally, we did not find any association between cytokines and endocannabinoids during IFN-a treatment. However, we found that six months post-treatment, AEA levels were negatively associated with IL-2 and IL-17a, and, at a trend-level significance, with IL-6 and IL-10. Interestingly, we also found negative association between AEA and IL-2 at baseline. One possible explanation could be that IFN-α treatment, because of its strong immune effects, may have masked such associations. We know from the previous studies that activation of the immune system can increase eCBs, which in turn are capable of reducing the inflammatory response (Abidi et al., 2018, Patsenker et al., 2015). For example, AEA was shown to inhibit IL-2 production in the peripheral blood mononuclear cells of the HCV patients (Patsenker et al., 2015). In another study, AEA supressed proliferation of human peripheral T cells, stimulated with CD3, CD19 and CD28 antibodies via CB2 receptors. It was suggested that AEA acted through the inhibition of DNA synthesis (Cencioni et al., 2010, Schwarz et al., 1994). Furthermore, AEA inhibited IL-2 production, a key growth factor in T cells proliferation and supressed the release of pro-inflammatory cytokines, TNF-α, IFN-γ and IL-17, which are part of the TH1 response (Cencioni et al., 2010). Indeed, in our study we observe that both eCBs increase during IFN-a treatment but only AEA remains elevated six months post-treatment, whereas 2-AG returns to baseline levels. This would suggest a bi-directional association between inflammatory processes and circulating AEA which occur six months post IFN-α treatment.

In our sample we found that HCV patients showed significantly lower baseline serum AEA levels compared with healthy controls, however there was no difference in serum 2-AG levels across the two groups. This is inconsistent with previous research showing elevated plasma levels of AEA and 2-AG in HCV patients compared with the control group (Patsenker et al., 2015). Strong evidence from the research on stress, suggests that chronic stress exposure attenuates eCB activity and reduces circulating eCBs (Yi et al., 2016). Living with the devastating, chronic condition such as HCV infection means that these patients are more likely to be exposed to higher levels of stress for a long period of time due to the nature of the disease itself. However, measures of stress were outside the scope for this study. Of note is that the majority of patients in our sample had HCV genotype 3, which could have also affected baseline immune responses, and subsequently eCB levels.

One of the limitations of this study is the sample size which was smaller than originally intended. Recent changes in NHS England affected the number of patients receiving IFN-α treatment and a large proportion of patients were treated through clinical trials. Additionally, early discontinuation of treatment and baseline comorbidities reduced the sample size, and the retention rate was 60% at six months follow-up. Therefore, we may have been underpowered to detect associations between eCBs and depression. Also, we did not collect data that were likely to influence circulating eCBs, such as exercise and food intake. Controlling for these factors would be the recommended next step in the future research.

Although IFN-α treatment is no longer a standard treatment for HCV, the results from this study provide a valuable insight into the role of peripheral eCBs in response to the immune challenge, suggesting their involvement in the immunomodulation. Future studies are needed to confirm whether these findings extend beyond HCV sample and IFN-α treatment. Although interpreted with caution, together with the findings from previous studies, our study provides further evidence that targeting eCB signalling may be of interest as an anti-inflammatory treatment in the future.

## Declaration of Competing Interest

Dr Forton has received speaker consultancy fees from companies that market drugs to treat hepatitis C, including AbbVie, Gilead, BMS and Janssen. He has received funding for trials from Merck. Dr Kosh Agarwal has received fees from companies that market drugs to treat Hepatitis C, including AbbVie, Gilead, Astellas, Intercept, Janssen, Merck and Achillon, and has received grants from AbbVie, Gileas, BMS and Roche. Prof. Pariante. Dr Mondelli and Dr. Zunszain have received research funding from Johnson & Johnson as part of a program of research on depression and inflammation, and research funding from the Medical Research Council (UK) and the Wellcome Trust for research on depression and inflammation as part of two large consortia that also include Johnson & & Johnson, GSK and Lundbeck. Dr Mondelli is also funded by MQ: Transforming Mental Health (Grant: MQBF1). The other authors declare that they have no known competing financial interests or personal relationships that could have appeared to influence the work reported in this paper.
