# Supplementary figures and images for: Differential effect of interferon-alpha treatment on AEA and 2-AG levels
Source: Brain Behav Immun. 2020 Nov;90:248–58. doi: 10.1016/j.bbi.2020.08.024 (PMC7575143; doi:10.1016/j.bbi.2020.08.024)

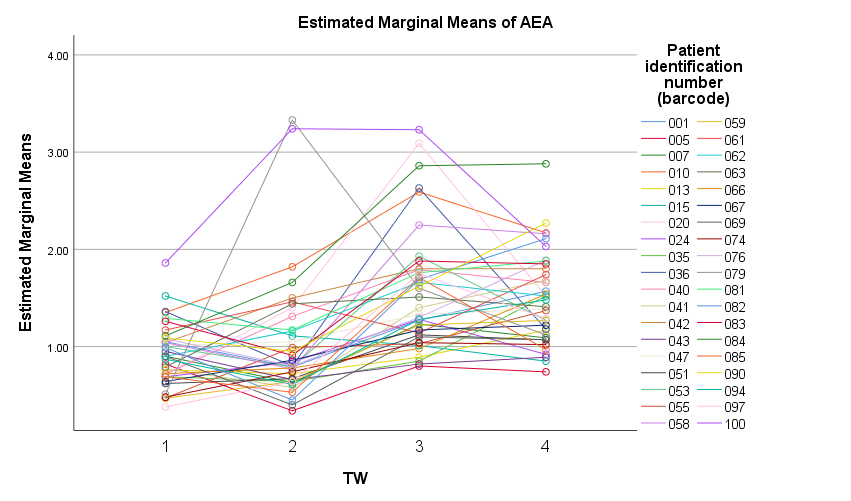


Legend

TW 1, 2, 3, 4 correspond to TW0, TW4, TW24 and FU, respectively


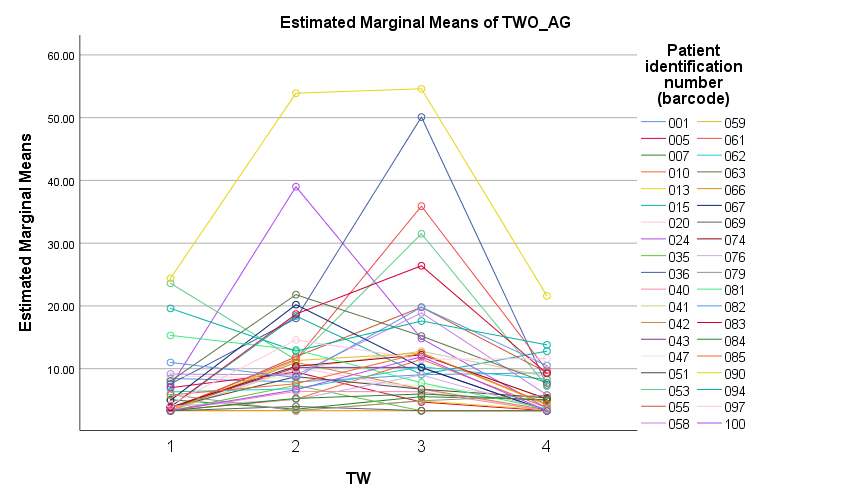

Supplement: Supplementary data 1 [file mmc1.docx]

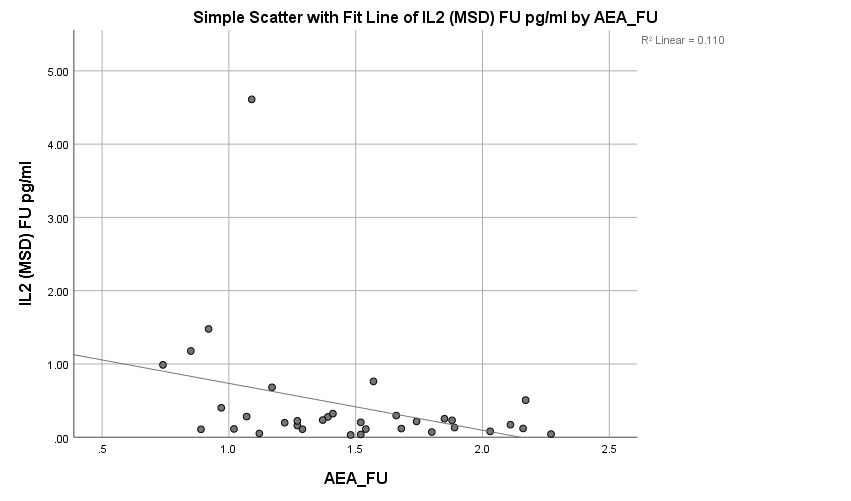


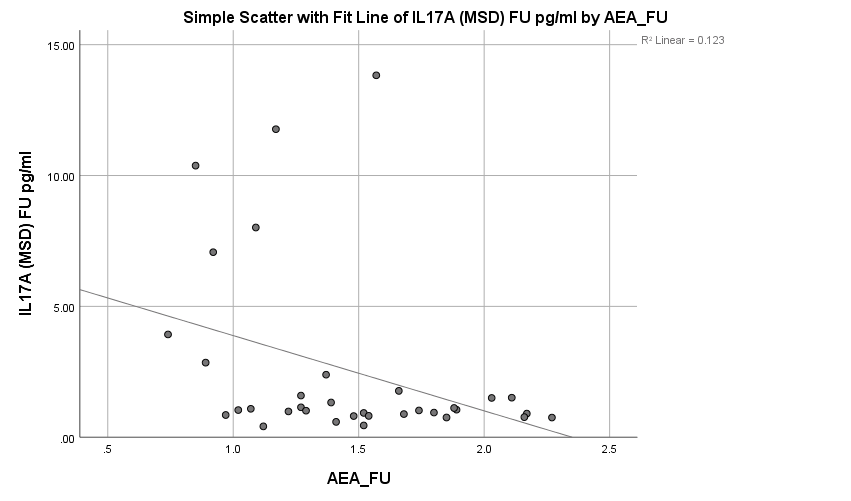

Supplement: Supplementary data 2 [file mmc2.docx]
